# Bodily Abounds. Hilary Mantel’s *The Mirror and the Light *as Cixousian "Feminine Text

**DOI:** 10.12688/openreseurope.20790.2

**Published:** 2026-04-10

**Authors:** Ante Andabak

**Affiliations:** 1 Faculty of Humanities and Social Sciences, University of Zagreb, Zagreb, 10000, Croatia

**Keywords:** Hilary Mantel, The Mirror and the Light, Hélène Cixous, écriture feminine, body words

## Abstract

One of the most critically acclaimed and successful examples of historical/political novels in this century, at least in the Anglophone world, is Hilary Mantel’s towering Thomas Cromwell trilogy, made up of *Wolf Hall* (2009), *Bring Up the Bodies* (2012) and *The Mirror and the Light* (2020). Mantel took the period and place done to death, the Court of Henry VIII, and imbued it with a new life and striking immediacy thanks to her decision to tell the story through the perspective of a blacksmith’s son from Putney, who became a Chief Minister, which in turn allowed the class aspect to take a centre stage in her writings on Tudors and held court where it usually rarely did. This paper, however, focuses on the way gender plays a role in this men-dominated world, where women notoriously fared extremely badly, but it will not do so by focusing directly on the dazzling female characters Mantel gives us, with, among others, Anne Boleyn and Jane Rochford, or by merely pointing out Mantel’s own gender. Rather, it can be argued that the rich vein of feminism resides in the indomitable style of novels. This will be done with the help of Hélène Cixous’s seminal concept of *écriture féminine*, and especially the so-called *body words.*

## Introduction

The first section explains how Mantel revitalised the genre of historical fiction by, among other things, making her trilogy double as political novels. The second section shows how the author, greatly concerned with historical accuracy, managed to write feminist novels without being anachronistic, the accomplishment even more extraordinary given her male main character through whose perspective the entire story is told. After determining how Mantel’s particular, corporeal style of writing made that possible, the third section will explicate Hélène Cixous’s conception of *feminine writing* as a theoretical approach fitting the material. The fourth section will return to *The Mirror and the Light* (the final novel in the trilogy, which this paper focuses on) and briefly show how Mantel’s metaphors are materially realised and embedded in the narrative. The fifth section tries to conceptualise Cixous’s idea of *body words* (embodied style of writing which is specifically *feminine*) with the help of John T. Hamilton’s philology of the flesh. Sections six and seven then explicate concrete examples from the novel. In the conclusion, it is argued that there are many more body words in the novels, even among the quotes appearing in this paper, than the two instances which this paper primarily focuses on.

## 1. Properly Political Historical Fiction

Hilary Mantel’s terrific and towering Tudor trilogy – made up of *Wolf Hall* (2009), *Bring Up the Bodies* (2012), and *The Mirror and the Light* (2020) – stands as a prime example of modern historical fiction, which is at the same time thoroughly political. There are few, if any, comparable recent titles in the Anglophone world in terms of both the critical acclaim and commercial success that these novels have garnered.

Mantel took the period and place done to death, the Court of Henry VIII, and found so much vivid life in it, which she then carefully and sumptuously recreated, that even the intellectual offspring of Jacobins, who do not have the stomach for the idea of royalty or taste for their intrigues and dramas, found themselves engrossed. She managed that outstanding feat by choosing to inhabit and tell the story entirely from the perspective of Thomas Cromwell, the most unusual historical figure who, despite having the lowest possible origins, rose to become Henry’s chief minister and his most important, trusted and powerful advisor.

Historiography and broader culture have consistently portrayed him as a scheming, conniving, Machiavellian knave with hands more smeared than the butcher’s, due to whose malign influence some great men like Thomas More were executed (most famously in Robert Bolton’s 1960 play *A Man For All Seasons*, adapted into a 1966 Academy Award best picture-winner of the same name). Such a unanimous verdict could be ascribed to historians and artists too often emulating the standard behaviour of kings after a rebellion in some part of the realm has been quashed: “the gentry pardoned, and the poor dangling from trees,” as was wryly summarised in *The Mirror and the Light* ( 1 on page 301). Not taking that well-trod road, Mantel decided to give Cromwell a fair hearing, and in the process, historical fiction with a bite and class consciousness unusual for the court-centric iteration of the genre was created.

Admittedly, some important historians already revealed the other side of the perennially tarred Cromwell, like G. R. Elton who had established him “as a statesman of the first rank” ( 2 on page 382) and Mary Robertson [ 1] whose “doctoral thesis on Cromwell’s ministerial household enabled Mantel to turn her belief that Cromwell was not just a villain but also a politician of genius into the basis for a fully realized picture of his life and times.” ^3^ But none of them filtered down to numerous fictional portrayals of Henry VIII and his cut-throat retinue. “Blacksmith’s boy to Earl of Essex – how did he do it? The story seemed irresistible. I thought someone else would write it.” ( 2 on page 382) She wanted to set it down on the page by the late 70s, immediately after writing her first and painstakingly precise novel – *A Place of Greater Safety* (completed in 1979, but published in 1992) – which centred on the lives of revolutionaries Georges Danton, Camille Desmoulins and Maximilien Robespierre. However, when she came around to it some thirty years later, still “no one had told the story of the man” who was one of the principal drivers of English Reformation, and the main architect of the annulment of the king’s first marriage to Katherine of Aragon; the resulting split from Rome, marriage to, and subsequent trial and execution of Anne Boleyn; and the dissolution of monasteries among many other events with great historical implications.

In Mantel’s ( 2 on page 383) interpretation, “Cromwell is an arch-plotter, smarter than Henry though not meaner.” While her sympathetic portrayal of him has been challenged ^5^ and the author, raised Roman Catholic herself, was accused of flagrant anti-Catholic bigotry, ^6^ no one would dispute her estimation that “there seemed no limit to [Cromwell’s] massive, imperturbable competence.” ( 2 on page 383) The crucible in which the modern English state has been formed had been handled in such a decisive and far-reaching fashion by the blacksmith’s son, whose varied ministerial activities all went into bringing about “a new conception of sovereign nationhood.” ( 4 on page 150)

His base, commoner background, opened the door for the class aspect to hold court where it usually never did, in the novels primarily devoted to the politicking of blue-blood personages. This was why someone like Mantel, who initially wrote with singular relish and passion about the French Revolution, could apply the same ardour to royals and high nobility long before the gleaming reflection bouncing off the guillotine blade could have shed the properly politically revealing light on the aristocratic goings-on.

In addition, her narratological choice to tell the story in the present tense and entirely through Cromwell’s point of view through a strictly focalised third-person perspective brought sensibilities of literary modernism to a genre of historical fiction that – despite some notable outliers that could come under the heading of historiographic metafiction [ 2], ^7^ – was usually welded to traditionalism of all kinds, not least the literary ones. “It has mostly been a conservative, nostalgic art form, prone to flatter the reader by embellishing the received version of events, and to soothe the reader, by taking the politics out of the past. The counterforce is real history—messy, dubious, an argument that never ends.” ( 2 on page 270)

Despite the ideal setting for “a certain kind of historical fiction that feeds collective fantasy” and “taps into that common childhood daydream that we are not the children of our parents but of more distinguished strangers, who will turn up any day to collect us,” Mantel rather plunges us into a painful past rife with politics. Due to the widespread “slavish, oily royalism of the genre” ( 2 on page 269), back when Mantel was starting out, “historical fiction wasn’t respectable or respected. It meant historical romance. If you read a brilliant novel like Robert Graves’s *I, Claudius*, you didn’t taint it with the genre label, you just thought of it as literature” ( 2 on page 262). But even as recently as the time of the publication of the first two instalments of her trilogy, multiple major critics who highly praised the novels were quick to point out how the “hackneyed label” ^9^ of historical fiction – which functions as something of “a byword for middlebrow wasteland” [ 3] – doesn’t fit them, with one reviewer marvelling at how the novels belonging to “a somewhat gimcrack genre not exactly jammed with greatness” could end up being so good and successful, and concluding, half-jokingly, how one of the reasons “is that Mantel seems to have written a very good modern novel, then changed all her fictional names to English historical figures of the fifteen-twenties and thirties.” ^10^

For this infusion of stylistic modernism in the still mostly medieval setting to work, though, the single consciousness through which all the events were to be refracted had to be someone special, and here we can point out the splendid congruence between form and content. Just as Cromwell was the greatest political supporter of the Protestant efforts to have the Bible translated and published in contemporary English, Mantel chose to write about the first four decades of the sixteenth century in modern vernacular and not to get caught up in the period-appropriate thous and thines. And just as Cromwell was one of the people who ushered in early modernity in England by spearheading the break with the traditional Papal authority, Mantel chose a narrative strategy stemming from and reliant upon a similarly consequential early 20th-century literary supersession of the 19th-century realist modes of representation [ 4].

## 2. The Non-Anachronistic Feminist Historical Fiction

We will no longer linger on these well-established points about Mantel’s Tudor trilogy, ^12^ but will rather take a stab at fleshing out the role gender plays in this feverishly patriarchal world where women in general – and King’s wives most publicly – fared extremely badly. The novels are chock-full of scenes that harrowingly capture what it was to be a woman, which is to say, regarded primarily as a birth-giving machine, with the concluding entry having an entire chapter titled “Broken on the Body” dedicated explicitly to it. It recounts Henry’s third wife, Jane Seymour, giving birth to a future short-lived king, Edward VI, then dying of postpartum complications twelve days later, and begins with this salvo:

What is a woman’s life? Do not think, because she is not a man, she does not fight. [...] her theatre of war is the sealed room where she gives birth. She knows she may not come alive out of that bloody chamber. [...] If she dies, she will be lamented and forgotten. If the child dies, she will be blamed. If she lives, she must hide her wounds. [...] Whereas we bless an old soldier and give him alms, pitying his blind or limbless state, we do not make heroes of women mangled in the struggle to give birth. If she seems so injured that she can have no more children, we commiserate with her husband. ( 1 on page 507) [ 5]

Sometime after this, with the next wife, Anne of Cleves, already in tow, Henry was still short of a few more male heirs that he felt were his due, and was, as usual, thinking about siring a son, which led to this elegantly accomplished novelistic intersection of class and gender.

‘Cromwell, could you have a child?’
He is startled. ‘I think you could,’ Henry says. ‘You are of common stock. Common men have vigour.’
The king does not know they wear out. At forty a labourer is broken and gnarled. His wife is worn to the bone at thirty-five. (768)

These two excerpts are hopefully enough to intimate what a treasure trove these novels are for those seeking to apply a gender lens to them. However, we will not be doing so here. Instead of simply focusing on the level of representation – as we did in the previous two examples – in what follows, we will deal with the gender of writing as is specifically realised through the stylistic characteristics of *The Mirror and the Light.* Everything we read passes through Cromwell’s consciousness, who, though no blatant misogynist and quite progressive by the appalling standards of the day, is a far cry from a fighter for women’s liberation, in no matter how embryonic a form. Given that fact, if what Christopher Hitchens said about *Wolf Hall* in a backhanded compliment were true *,* namely that “You’d never know it was written by a woman”, we would have nothing but Mantel’s own gender to go by. ( 13 quoted on page 129)

Alaa Alghamdi was the first to take up the question of whether Mantel’s trilogy (at the time, still without the concluding instalment) belongs to *women’s writing* and answers in the affirmative, crediting precisely this dimension of her writing for revivifying and even redefining historical fiction. He helpfully distinguishes between form and content as separate spheres where this gender question needs to be posed, and urges that, beyond the portrayal of female characters, “a more detailed examination of the role of gender within the narratives will include, as well, the way that they are written.” ( 13 on page 130) Unfortunately, he does so only briefly, while also seemingly equating embodiment and intuitiveness with *women’s writing*, which is uncritically glossed as “woman’s way of knowing the subject”. ( 13 on page 119)

Instead of *women’s*, we will emphasise how Mantel’s is the *feminine* form of writing, one *not* bound by or issuing from any single gender identity. Rather, it is the expressive mode which calls into question the rigidity or even advisability of such straightforward identifications. This allows the text, consistently restricted to the perspective of the male character and mostly dealing with other powerful men, to be fully feminist at the same time. Let us consider the following masterful state-of-the-nation snapshots as an example:

The common folk of England live on songs and tales and alehouse jokes. Spending their pence on candles to burn before holy images, they live in the dark, and in the dark take fright. Let us say a calf is born dead. By the time the tale crosses a field, it is a calf with two heads. Cross a stream, and it is a calf with two heads, chanting backwards in Latin, and some friar is charging a shilling for a charm against it. So it goes, in half a day, from abortion to Antichrist: and somehow, everybody is poorer except the priests. (322)

In this passage, Cromwell’s disdain for the avaricious clergy and his proto-enlightenment impulse of wanting to end the illiteracy and ignorance of the broader population are on full display. The translation of the Bible into English and other spoken languages was intended to abolish the middlemen between God and believers, which explains why it was a preeminent political question. In addition, if the word of God became available in the common vernacular, denying large swathes of the population rudimentary education would become tantamount to barring them access to Christ.

While the thoughts recorded are of Cromwell, the character, as there are no narratorial asides or metalepses in the novels, their virtuoso renderings in sentences are decidedly authorial, without being anachronistic. Indeed, while all the references could conceivably have been made by the historical Cromwell, their specific wordings are not even of a piece with him as the character created by Mantel. By claiming this, we are entering a contentious terrain that one of the key texts of modern literary criticism, Roland Barthes’s 1967 essay “The Death of the Author”, strikingly delineated with its opening paragraph:

In his story *Sarrasine* Balzac, describing a castrato disguised as a woman, writes the following sentence: ‘ *This was woman herself, with her sudden fears, her irrational whims, her instinctive worries, her impetuous boldness, her fussings, and her delicious sensibility.*’ Who is speaking thus? Is it the hero of the story bent on remaining ignorant of the castrato hidden beneath the woman? Is it Balzac the individual, furnished by his personal experience with a philosophy of Woman? Is it Balzac the author professing ‘literary’ ideas on femininity? Is it universal wisdom? Romantic psychology? We shall never know, for the good reason that writing is the destruction of every voice, of every point of origin. Writing is that neutral, composite, oblique space where our subject slips away, the negative where all identity is lost, starting with the very identity of the body writing. ( 14 on page 142)

The reason why we decided to reproduce this paragraph in full is that, even as Barthes is analysing the problem of assigning any aspect of literary text, irrespective of its subject matter, to some undisputed instance (paradigmatically, that of the Author), the impossible-to-untangle narratological problem at hand just happens to be thematically woven around the troubling notions of womanhood. “Is it an accident that Barthes selects a fragment from the semantic code which refers to *femininity* as his example of disoriginating discourse?” Liedeke Plate asks, and then continues with another pointed question: “And why this indifference to the subject of the utterance, [...] at the very moment the woman writer is coming into her own?” ( 15 on page 162)

We will not pursue these questions further, but ours is also a feminist reason, albeit a different one, for going against Barthes. He advised against trying to parse what belongs to the character in the text, and what belongs to what he termed the *scriptor* – i.e., not “the Author” who is “antecedence to his work as a father to his child” but that writing instance which is “born simultaneously with the text,” and is “in no way equipped with a being preceding or exceeding the writing[.]” ( 14 on page 145) However, in our case, finding the specific spaces marked by scriptor within the text is the way to grasp and appreciate Mantel’s elegant and quicksilver way of dealing with the great worry about historical fiction she herself eloquently posed: namely, when it comes to writing “about the victims of history,” whether we are “reinforcing their status by detailing it.” The other available option is to rework the history so that the victims could become not just moral, but actual victors. “This is a persistent difficulty for women writers, who want to write about women in the past but can’t resist retrospectively empowering them. Which is false.” ( 2 on page 272–3)

Though some of the most intelligent, witty and interesting characters in these novels are women, with Anne Boleyn and Jane Rochford as particular nimble-witted conversationalist highlights (with their being depicted so not a case of contemporary posthumous empowering, comporting, as it does, with historical facts), Mantel, however, resists the urge of surreptitiously indulging in the revisionist come-uppances to fictionally right the historical wrongs. For her, that is simply a mistaken track for the genre she is writing in, because to compose a work of historical fiction requires being profoundly respectful of the past, not as “a rehearsal” but “the show itself”, and consequently, being committed to understanding our ancestors not as “us in an unevolved form”. ( 2 on page 273) So it is in the indomitable style of her novels – in the nooks and crannies solely occupied by the scriptor, and containing phrases like *so it goes … from abortion to Antichrist* – that the richest vein of feminism in these texts can firmly reside, without undermining historical veracity or wallowing in exploitative depictions of cruelties past.

## 3. Cixous and the Gender of Writing

In claiming that Mantel’s style is *feminine*, and therefore, *feminist*, we are, of course, taking a cue from Hélène Cixous and her seminal conception of *feminine writing* ( *écriture féminine*) as one thoroughly marked by the bodily. She advocated in her ebullient 1975 essay “The Laugh of the Medusa” (“Le Rire de la Méduse”) for women to start writing and speaking out without customary feelings of shame and inadequacy. “It’s not to be feared that language conceals an invincible adversary, because it’s the language of men and their grammar. We mustn’t leave them a single place that’s any more theirs alone than we are.” ( 16 on page 887)

Writing, which is unabashedly feminine for Cixous, must be written through women’s bodies and must embrace the long-suppressed and denied but impossible-to-snuff-out materiality in language. Such texts would be those which “are very close to the voice, very close to the flesh of language, much more so than masculine texts” and she conjectures that’s so because “they don’t rush into meaning, but are straightway at the threshold of feeling. There’s tactility in the feminine text, there’s touch, and this touch passes through the ear.” ( 17 on page 54)

Her brand of feminism could initially strike an inattentive reader as hopelessly essentialist, yet when she singles out what authors in France of that time she takes to be feminine writers she gives three names: “Colette, Marguerite Duras, ... and Jean Genet” ( 16 on page 879), making clear it’s not the biological sex which determines the gender of writing, it’s taking up topics and approaches to language which were previously dismissed out of hand as irrelevant and inappropriate.

So, despite all the potentially misleading talk of writing in “white ink” of “mother’s milk” ( 16 on page 881), the intended and the actual effects of Cixous’s politico-stylistic laughing-Medusa intervention weren’t essentialist, but exactly the opposite.

In fact, Cixous’s text played a major role in displacing the debate from feminism to gender. Her conception of sexual difference not as an effect of biology but as a matter of libidinal economy, and specifically a question of different practices of the relationship between self and other, was an important factor in the shift of emphasis from ‘women’s studies’ to ‘gender studies’ that took place in the late 1970s and early 1980s. ( 18 on page 157)

Additionally, Mairéad Hanrahan reminds us that Cixous “broke new ground both in valorising femininity and in proposing that it was not the exclusive property of females.” ( 18 on page 157) Still, it could be argued that with her take on femininity, she uncritically accepted, though she radically revalued, the already held and imposed ideas about women, principally, that they are more bodily determined than men. According to Ngũgĩ wa Thiong’o ( 19 on page 21), that type of mistake crept inside the artistically glorious and historically immensely important Négritude movement, because “its theoretical articulation could be mystical in its metaphysical abstraction, as when [Léopold Sédar] Senghor tried to explain the difference between black Africa and the West in the mellifluous metaphysical nonsense of his statement *Emotion is Negro as Reason is Greek.*” [ 6]

This is no accidental parallel, either, as Cixous treats “femininity as a repository of tropes of embodied alterity and otherness, whether in terms of race, class, ethnicity or gender,” and “formulates a liminal politics whereby the marginalized and the excluded can function as an antagonist force opposing colonialist and patriarchal logocentrism.” ( 21 on page 3) Considering Cixous’s psychoanalytic background, it’s unsurprising that she frequently riffs on Freud’s ( 22 on page 35) infamous assertion that “the sexual life of adult women is a ‘dark continent’ for psychology” where Freud left “the metaphor of the ‘dark continent’ in its original English” ( 23 on page 49), unambiguously tying it to the reason why it gained wide currency – the colonial explorer H. M. Stanley’s 1878 account of the search for the sources of the Nile titled *Through the Dark Continent.*

One of the most powerful moments in Cixous’s justly celebrated essay is when she uses this trope to combine fights for black and women’s liberation. Speaking of girls and women, she noted:

You can incarcerate them, slow them down, get away with the old Apartheid routine, but for a time only. As soon as they begin to speak, at the same time as they’re taught their name, they can be taught that their territory is black: because you are Africa, you are black. Your continent is dark. Dark is dangerous. You can’t see anything in the dark, you’re afraid. [...] And so we have internalized this horror of the dark. ( 16 on page 877–8)

But Cixous does not want to accept this classical view of the world; rather, she proudly states, “we the repressed of culture [...] we are black and we are beautiful.” It is crucial to make clear that Cixous did not at any point leave reason to men, explicitly lambasting writing “which either obscures women or reproduces the classic representations of women (as sensitive-intuitive-dreamy, etc.).” ( 16 on page 878) She just saw women as logical allies to other denigrated concepts in binary oppositions – mind/body, light/dark, spirit/matter, activity/passivity, and many others – on which dominant cultures built their sense of worth.

Rallying for and delving into the misunderstood and maligned is the preservation of genuine feminine text, and though it may seem like falling into the trap of internalising stereotypes if feminine writing is marked by a great focus on corporeality, it is, in reality, unreasonable and dead wrong to uphold the opposition between body and mind in the first place.

Rather than holding up the banner of naive biologism and sex-centric political separatism [ 7], Cixous can even be seen as an avant-garde and a poetic precursor of sorts for the paradigm that would soon after her essay, and completely unrelatedly to it, come to sweep the fields of cognitive linguistics and sciences – embodied cognition. ^25^^,^^26^ While not explicitly feminist, this approach to meaning-making as grounded in bodily experience ties in nicely with Cixous.

## 4. Understanding Metaphors Materially: The Mirror, the Light, and the Fires

Language was traditionally understood to belong to the domain of spirit and culture; our ability to speak was seen as a clear distinguishing mark between high-minded humans and animals regarded as mere bodies and instinctual automatons. Moreover, cognition as a phenomenon in which language is the primary and privileged means of expression, regardless of who or what is thinking (whether human or machine), was supposed to be separate from the substrate (whether organic or mechanical), which made that thinking possible. “Cognitive scientists were interested in cognition regardless of its physical instantiations just in the way that one could try to characterize flying regardless of whether it was in the context of jets, birds, butterflies, or paper airplanes.” ^27^ Experimental research has shown that, on the contrary, cognition is completely suffused with corporeality and submerged in flesh, and that our embodiment is deeply embedded in languages and crucially shapes what is thinkable and sayable.

Poets have implicitly known that for a very long time, with so many of our phrases frozen metaphors, waiting for the right verse to be thawed and brought back to life, to physical interactions, to friction. It’s what makes the language tick and be lyrical, for there to be “tussle in every line” instead of “no contest at all, just a smooth surrender to idiocy” (192), as Cromwell thought to himself, comparing the lines of Thomas Wyatt, an ambassador and a foremost poet of his time to whom he acted as a patron, with that of a far lesser verse-maker whose private love poems have come to his attention for political reasons.

Mantel herself excels at making a scintillating use of the most run-of-the-mill idioms, like “there’s no smoke without fire”, and bringing it back to the threatening reality of sulfuric blaze (167). The entire novel is alight with fires, both metaphorical and increasingly literal, in which, to Cromwell’s disgust and dismay, purported heretics burn. As a Reformationist and humanist, he wants to see the Church of England shift away from such grisly Roman practices as a matter of principle. However, there is more to it; he fears it as his own fate.

When watching the gloaming, “the sun a perfect crimson orb above the line of the downs,” he thinks of those who died by fire, “as if they have fallen into the sun,” among them Tyndale, the first translator of the Bible into English, who was burned at the stake for Lutheran heresy in the Netherlands. (575–6) The sun was also the reason for the fall of Icarus, and Cromwell sees himself as a Daedalus of sorts. He poignantly muses why we only blame the mythical ancient inventor “for the fall, and only remember his failures? He invented the saw, the hatchet and the plumbline. He built the Cretan labyrinth.” (156) After one of his theologian friends, John Lambert is burned to death for denying the real presence of Christ in the Eucharist, the vague forebodings and foreshadowings from the first half of the book give way to explicit fear. Cromwell confides to his closest ideological ally, Thomas Cranmer, archbishop of Canterbury and religious leader of the English reformation: “If the king can burn this man, he can burn us.” (615)

That is the same Henry who, throughout the novel filled with scathing humour, is the resident butt of a joke: “Once the king had grasped what he was being told, he had shouted at the top of his voice that the business should be kept quiet.” (189) The king behaves like a sulky child, enamoured with the sight of himself, with mirrors – of which he “owns more than a hundred” (629) – and fine clothes as favourite things to busy himself with (424), prompting Cromwell to think to himself, before Henry becomes “petulant” because he hasn’t been complimented on his costume, that “there is a limit to how much awe a man can feign.” (203)

Nonetheless, Cromwell is more than capable of high praise, as when he says, “Your Majesty is the only prince. The mirror and the light of other kings.” He takes to it, repeating the phrase, “as if cherishing it”, loving, as he does, to have his ego stroked. (544) In that piece of flattery, a multitude is contained.

For one, contextually speaking, it was still a time of *specula principum*, a medieval literary genre of didactic political writing, intended to be instruction for monarchs on how to rule. Cromwell, who has read “a library of those volumes called Mirrors for Princes” (811), might as well have said, in modern parlance, that Henry was a textbook example of how to be a king. However, this historically appropriate and much more poetically inspired phrase could, and indeed did serve as a title and running metaphor for the entire book, which begins with the beheading of Anne Boleyn with the blade incised, as we later find out, with the Latin words of a prayer: “Mirror of Justice. *Speculum justitiae.* Pray for us.” (188) The mirrors and glass in the story multiply henceforth, carrying an ominous meaning. Cromwell imagines Anne Boleyn in “some draughty hall of the hereafter, where the walls are made of splintered glass.” (198) Ironically, Cromwell knows that the king “wishes to be known as Henry, Mirror of Justice” (388), and right after he calls him the mirror and the light and they continue with a friendly conversation while he senses “the dead queens blink at him, from behind their broken mirrors.” (544)

After Lambert’s death by burning, Cromwell thinks about his own precarious position: “If Henry is the mirror, he is the pale actor who sheds no lustre of his own, but spins in a reflected light. If the light moves he is gone.” (617) There’s something else, however, that a mirror or some other type of glass can do with light in the right circumstances, and that is to start a fire. That third entity, absently present in the title, and never spelt out elsewhere, nevertheless lingers and permeates the novel, like the heavy smell of a human being “reduced to bone, rendered to fat, to paste”, which Cromwell, as a boy, saw up close and even breathed in. Indeed, “some made way, thinking it a pious work to show a child a burning” (557), enabling him to witness first-hand the execution of an eighty-year-old Joan Boughton, accused of being a Lollard, the religious group from England who were followers of John Wycliffe. Immediately preceding Protestantism, Wycliffe likewise wanted the vernacular Bible, opposed the official Church and didn’t believe in the doctrine of transubstantiation.

## 5. Body Words and the Philology of Flesh

Transubstantiation – the doctrine according to which Christ’s flesh and blood are in fact, and not merely metaphorically, present in the Eucharist – allows us to go straight back to Cixous who, with her notion of “body words” ( *corps du mot*), plays on that grand prologue from the Gospel of John proclaiming that the God’s “Word became flesh and dwelt among us” (John 1:14 [KJV]). Christ as “the Word Incarnate ( *logos ensarkos*)” can in literary and non-theological terms be grasped as “the means and the end, the medium and the message” being inseparably bound ( 28 on page 46). Such indivisibility lends itself well to being read as a forceful repudiation of “the history of philosophy [which] has tended to divorce language from thought” in an effort to arrive at the latter in the purest possible form, the tack which has stubbornly persisted even in ostensibly non-metaphysical, empirical cognitive sciences until most recently. Johannine understanding stresses that “a word cannot dispose of its verbal medium” ( 28 on page 19) but must rather dwell bodily in it to allow for the deliverance of meaning. Simply put, no sense is to be made of the work away and apart from its concrete, sensuous wording; just like there’s no path to God except through Christ – “I am the way, the truth and the life” (John 14:6 [KJV]) – given that it can, quite literally, happen only over his dead body.

For Cixous, this broader incarnational logic can be readily observed in the flesh whenever one listens to “a woman speak at a public gathering” because it’s not that she so much speaks as much as “she throws her trembling body forward” letting go of herself, flying, as “all of her passes into her voice,” so that “it’s with her body that she vitally supports the ‘logic’ of her speech. Her flesh speaks true. She lays herself bare. In fact, she physically materializes what she’s thinking; she signifies it with her body.” ( 16 on page 881)

The paragraph in which “body words” explicitly appear in “The Laugh of the Medusa” is dedicated to psychoanalytic patients who were at the beginning mostly women and who “have furiously inhabited” their “sumptuous bodies” with Cixous praising them for being “admirable hysterics who made Freud succumb to many voluptuous moments impossible to confess, bombarding his Mosaic statue with their carnal and passionate body words, haunting him with their inaudible and thundering denunciations, dazzling, more than naked underneath the seven veils of modesty.” [ 8] ( 16 on page 886)

His arguably best-known analysand, Dora – a woman who suffered from aphonia and who, after initial success, broke off analysis due to interpretative disagreements, much to his chagrin ^30^ – Cixous directly addresses with “you the indomitable, the poetic body, you are the true ‘mistress’ of the Signifier.” ( 16 on page 886) She also wrote a powerful play about it, *Portrait of Dora* ^31^ based on this case study [ 9].

Body words cover the vast field in Cixous, from women analysands who have no choice but to speak with their bodies because the language in which they could be heard is systematically denied to them, to writing and speaking in a conventionally articulate language in such a way that its generally suppressed materiality is given a voice and made to resound boldly and bodily.

Body words in writing are not simply those denoting something corporeal, but the words used so as to expose the weight of physicality that has been covered up, the words which point out the bodily origin of something seemingly divorced from it. But, in our Cixous-informed conceptualisation, to qualify as fully-fledged body words, they must not only expose that we are all flesh (like the examples from the previous, fourth section, which materialised metaphors), but must also speak to the fact that only women get fixed in the corporeal and perceived solely through that lens (like the phrase *so it goes … from abortion to Antichrist.* It was, after all, traditionally put on women to shoulder the blame for the horror, unavoidable to all, of being vulnerable, needy, dependent, for the horror of being material and mortal. As Elisabeth Bronfen ( 33 on page 165) notes in *Over Her Dead Body: Death, Femininity and the Aesthetic*:

Woman, constructed by culture as man’s symptom, marks the site where repressed material resurfaces, materialises, returns. The feminine body is used to figure death as the repressed *par excellence* and the displacement of death on to the feminine allows a mitigated articulation of that value otherwise threatening to the stability of the system.

Feminine writing is attentive to the burden a body can be, to the germs of death and decay it inevitably carries; yet, it also insists on it as our exclusive avenue for everything, either good or ill, that we can experience or have. In theoretical terms, that would lead us, as Wendy Brown ( 34 on page 196) urges us to do, to conceptually start from

the *human animal* rather than the human mind or the human male. We need to discern how to be free as ourselves rather than free from what we are, what bears and sustains us. Above all this involves recognizing that the body is the *locus, vehicle, and origin of our freedom, rather than the encumbrance to it Western political theory always claimed the body to be.*

This deep philosophical insight is present not only at the content level of insightful feminist literary works but also in their very form. Writing feminine texts means holding fast to the materiality of the signifier and not allowing it to easily and smoothly give way to the signified itching to immediately overtake and overshadow it. This is how we can best understand the already quoted parts from Cixous ( 17 on page 54) on *feminine texts* not rushing into meaning, but abiding “at the threshold of feeling” and Mantel’s brilliant phrase about bad poetry doing the exact opposite with its “smooth surrender to idiocy” ( 17 on page 192).

Complementary to this, reading feminine texts entails not jumping to the import of the work without stopping to take in and appreciate the specific language that makes it possible. While writing feminine texts means writing through the body and using body words, reading such texts means engaging with what John T. Hamilton ( 28 on page 21) inspiredly called the philology of the flesh. He makes a persuasive case, based most directly on the philosophies of Maurice Merleau-Ponty and Jacques Derrida, for distinguishing “between the organized, functional body and the almost anarchic flesh.” ( 28 on page 17)

However, we decided to stick with the body words and bodily for the reason that Hamilton could definitely get behind – because of the repetition of the letters, because of the materiality of the signifiers, because it allowed us to say bodily abounds, what with the flesh, though the philosophically more accurate term, we would not have been able to do. The chief characteristic of the philology of the flesh is “a love for the word’s material features that contribute to but are ultimately irreducible to information.” This philology’s particular *philia* always has in mind and is driven by the “fleshly facticity” of any given language. Such practice of reading “exceeds subject-directed intentions and usages without neglecting meaning altogether. In this way, the philology of the flesh indulges in an oversaturation of meaning, which far outpaces the word’s denotative functions.” ( 28 on page 21)

## 6. “Gash” as a Body Word

Surfing on such an overflowing of the potential sense to be made of signifiers is what we will attempt to do now. Briefly starting with the part of the other sentence in “The Laugh of the Medusa” which has a variation of the “body word” in it, and comes from the same Dora paragraph we already covered the beginning and the end of, we will then go on to read a very particular twelve words from *The Mirror and the Light.*

Cixous ( 16 on page 886) talks of women “who, with a single word of the body, have inscribed the vertiginous immensity of a history which is sprung like an arrow from the whole history of men and from biblico-capitalist society,” and this, taken more literally than likely intended, can be perfectly applied to Mantel’s style of writing, especially as it skilfully coils and weaves around the biblico-capitalist themes, the latter in its inception, the former in one of its most fateful periods, the Reformation, with the controversy around the Bible front and centre.

Pondering what he and the rest of the reformationists have accomplished thus far in England, Cromwell feels assured for pecuniary reasons that their work would be safe even after Henry dies: “once the lands are given out, no subject will want to give them back to the church. Prayers may be rewritten, but not leases. Hearts may revert to Rome, but the money never will.” (272)

Abhorring the priests for dwindling the worse-off with the supposedly sacred objects and for being “full of money, screwed out of poor folk for pretended forgiveness of their sins” (532), Cromwell wishes to “break the shrines” to “found schools” and to “[t] urn out the monks and buy [...] alphabet books for little hands.” He wants to “draw out the living God from his false depictions,” and is confident that in a generation, “no one will ever believe we bowed to stocks of wood and prayed to plaster. The English will see God in daylight, not hidden in a cloud of incense; they will hear his word from a minister who faces them, instead of turning his back and muttering in a foreign tongue.” A staunch iconoclast, Cromwell wants the faithful to “cherish their Saviour in their inner heart, instead of gawping at him painted above their heads, like a swimming inn sign.” (272) This was not a simple matter of aesthetic preference but was of the greatest possible political consequence:

The Reformation practice of iconoclasm brought about the destruction of Catholic artefacts. Because these were associated with the communal life of the Church and were experienced visually, they embodied a public history that was collectively understood. This shared legacy was replaced by vernacular literature that made spirituality private and, in rejecting the pan-European language of Latin, asserted geographical identity. This enabled the modernizing English state to undermine the universal authority of Christendom. ( 35 on page 28)

Cromwell’s theological beliefs, though undoubtedly progressive when compared to those who “thought if you let the people read God’s word for themselves, Christendom would fall apart” and were of firm opinion that the poor “needed the constraint of ignorance” (429), nevertheless fit all the more easily into the traditional pitting of the cerebral against the corporeal. “When they can read the Bible for themselves, they will be closer to God than to their own skin. They will speak His language, and He theirs.” (448)

Cromwell wanted to implant the Holy Writ into people in its ideality – the only part of the text that can survive translation – so it would be deeper and more intimate than one’s body, one’s skin. Mantel’s feminine text – which proudly carries the opposite valence when it comes to corporeality – takes the opportunity to picturesquely revel in Cromwell’s iconoclast rancour inspired by painterly depictions of “simpering virgin saints with their greensick faces, and Christ with the wound in his side gaping like a whore’s gash.” (272)

The principal meaning of the word gash is a long, deep cut or wound, but it also metonymically serves as a vulgar term for female genitalia, as well as an offensive name for women collectively regarded in sexual terms. The passion of Christ, with its countless artistic renditions and interpretations, was inscribed with a whole new dimension when another meaning of gash was invoked in the whorls of red paint representing the thoroughly lacerated body. Cromwell, the character, meant it only as a well-placed insult at the expense of the pompous and gore-obsessed Catholic Church, but the text said so much more if we just pause and take a closer look at the neat biblical number of twelve body words composing it.

Painters of the crucifixion often emphasised evidence of suffering on Christ’s body to arouse horror and gratitude in us all for whose sins He so painfully died ( 36 on page 302–10). Likening the holy wounds of the most exalted body — indeed of the Word made flesh — to routinely abused and demeaned body parts of prostitutes who have always formed one of society’s most hated and marginalised groups was a stroke of genius. It allowed the long history of violent repression and vilification of women, regrettably still far from being past, to be, in a few words, deftly insinuated onto the figure who was the lynchpin of the religion whose members became one of the most ardent perpetrators of gynocide. The physical state of women who sell their bodies was subversively inscribed onto the Son of God, the one who deigned to pass the birth canal only if a hymen waited at the end.

Hilary Mantel wrote about the deadening effect of the cult of the Virgin Mary in her childhood: “Pray all you like, you are not going to be both a virgin and a mother; this was a one-off by the deity, a singular chance for sullied female flesh to make itself acceptable to the celibate males who were in charge of whether or not we got to heaven.” She also asked there “the old embarrassing questions” like – “When Jesus was delivered, what happened to Mary’s hymen?” Related concerns appear in *The Mirror and the Light* in the already mentioned “Broken on the Body” chapter, about Jane Seymour dying shortly after giving birth:

When Mary gave birth to her Saviour and ours, did she suffer as other mothers do? The divines have sundry opinions, but women think she did. They think she shared their queasy, trembling hours, even though she was a virgin when she conceived, a virgin when she carried: even a virgin when redemption burst out of her, in an unholy gush of fluids. Afterwards, Mary was sealed up again, caulked tight against man’s incursions. And yet she became the fountain from which the whole world drinks. ( 37 on page 508)

This passage discusses the perpetual virginity of Mary, the doctrine according to which her hymen was not broken, either before or after giving birth. However, given that the disputes about the admissibility of translating the Bible into languages other than Latin were one of the main historical factors in the Reformation period and consequently in the Cromwell trilogy, there is an astonishing irony we would be remiss not to point out: the whole sordid business with Mary being the virgin springs, at least to some extent, from the insufficiently accurate translation of the Old Testament Hebrew to Greek.

In The Gospel of Matthew, the reason why Mary had to be a virgin was because of what “the prophet Isaiah in the Jewish scriptures wrote, ‘A virgin shall conceive and bear a son, and his name shall be called Immanuel’ (Isa. 7:14). Matthew quotes this verse and gives it as the reason for Jesus’s unusual conception—it was to fulfil prophecy.” (Matt. 1:23) The Gospel writer “read the book of Isaiah not in the original Hebrew language, but in his own tongue, Greek”, that is, from the Septuagint whose translators rendered the original Hebrew word for ‘ *young woman* ( *alma*)’ – irrespective of whether she had sex – “using a Greek word ( *parthenos*) that can indeed mean just that but that eventually took on the connotation of a ‘young woman who has never had sex.’ Matthew took the passage to be a messianic tradition and indicated that Jesus fulfilled it, just as he fulfilled all the other prophecies of scripture, by being born of a ‘virgin.’” ( 38 on page 242–3)

## 7. “Travelling Bruise” as a Body Word

Finally, we return to the word gash. In the trilogy, it is used in its secondary, sexual sense only once, but it is present in its primary meaning of cut or slash throughout. It is there already, at the beginning, with *Wolf Hall,* which starts with the young Thomas on the floor, getting kicked nearly to death by his drunken father. The fifth sentence of the novel goes: “Blood from the gash on his head – which was his father's first effort – is trickling across his face.” ( 39 on page 3)

Right on the heels of that, Christ likewise makes an appearance, in the form of a curious insult: “‘Come on, boy, get up. Let’s see you get up. By the blood of creeping Christ, stand on your feet.’ Creeping Christ? he thinks. What does he mean?” ( 39 on page 4) These same sentences mark the end of the penultimate paragraph of the final novel; they are what goes through Cromwell’s head in the last moments before his execution (875).

When done correctly, insults are primed to stay with us; their adhesive power and the ability to get under our skin are unparalleled. Another popping up in *Wolf Hall* ( 39 on page 468) and then in *The Mirror and the Light* is the phrase “travelling bruise”, which is another instance of body words. While the feminist credentials of our previous body words were readily apparent, this one requires context for its radical resonance to be understood.

At one point early in the last novel, Cromwell’s particularly becoming clothes are enumerated, like “a green velvet coat,” “a riding coat of deep purple,” of the last, “a darkish crimson” robe, it is said by “one of Anne’s ladies” that he looks in it “like a travelling bruise.” (63–4) It might be objected that all our examples of body words are so obviously thematically linked to the flesh and that any formal, non-representational notions about them are empty talk. While indeed, they are all flesh-related, the only reason they were able to catch attention is by being so finely phrased on the level of signifiers. Insults often point to the bodily aspects of a person; yet, what makes them stick is not solely what they refer to; it is whether, once they do that, they have a ring to it.

Again, as with comments on Christ’s gaping wound, it was only meant as an insult, yet the travelling bruise doubles as a breathtakingly vivid and lyrical encapsulation of Thomas Cromwell’s life. “At fifteen he was on the road with his bundle, bruised and fleeing, heading for another bruising and another war; but at least, as a soldier of King Louis, he was paid to receive blows.” (67) Travelling and bruising through Europe, he gained knowledge and skills with which he fuelled his meteoric rise when he returned to England. But to the dizzying depths of meaning contained in the phrase “travelling bruise”, the final, devastating layer was added near the very end of the massive novel. Cromwell falls from grace and gets imprisoned and interrogated at the place where he interrogated so many, the Tower.

A touching, subtle commentary on Cromwell’s iconoclasm plays out there, when, in his cell, he tries to read Erasmus’s *Preparation unto Death*, but it “tires his eyes; he would rather look at pictures” so he takes his book of engravings and “sees Icarus, his wings melting, plummeting into the waves” yet Cromwell thinks now, triumphantly and defiantly, of the moment when the flyer “gained height” how “there must have been an instant” when Daedalus “knew, in his pulse and his bones, *This is going to work.* And the instant was worth the rest of his life.” (816)

To get back to the travelling bruise, during the interrogation, he gets told something seemingly laughable, that he was seen to wear “a doublet of purple satin” before he became a lord.

He does not laugh, because he sees where this is tending.
Norfolk demands, ‘What gave you the right to wear such a colour? It is the preserve of royal persons and high dignitaries of the church.’(818)

Thus, the dark hues he bore on his body and later draped himself in the finery of the same tones [ 10] became one of the ways to construe the guilt of the commoner who flew too high and too close to the sun. He was not burned like a heretic in the end, only axed, but no prayers were inscribed on it; there was no *speculum justitiae* (874). At least, he got light in the last moments, and after so much time, with Mantel’s incredible account, the new one shone on him.

Thomas Cromwell’s final thoughts are narrated with a blistering brio. After his head is cut off, in the very last split second of his consciousness, he looks

for the flitting, liquid scarlet. Between a pulse-beat and the next he shifts, going out on crimson with the tide of his inner sea. He is far from England now, far from these islands, from the waters salt and fresh. He has vanished; he is the slippery stones underfoot, he is the last faint ripple in the wake of himself. He feels for an opening, blinded, looking for a door: tracking the light along the wall. (875)

This is how *The Mirror and the Light* ends, with the addition of a closing epitaph, courtesy of Petrarch’s epic poem in Latin, *Africa*, “When the darkness is dispelled, our descendants will be able to walk back, into the pure radiance of the past.” (877) Mantel bathed Cromwell in that radiance, but what made her light so coruscating was the distinctly corporeal character of her writing.

## Conclusion

We have tried to conceptualise body words as distinct loci of femininity on the formal level of *The Mirror and the Light*, and to argue that this is the most potent source of the novel’s powerful, non-anachronistic feminism. It is important to highlight that, besides the specific body words analysed in the previous two sections, the text abounds with them, even in the passages we quoted for other purposes (for example, the phrase “caulked tight against man’s incursions” about Mary’s perpetual virginity).

On the other hand, and as we have already indicated, the examples from the fourth section – concerning the way Mantel rematerializes and plays with the metaphors surrounding mirrors, lights and fires – would not be body words. These metaphors – as well as assonance, alliteration and other poetic devices which prolong the hovering of the signifiers around the threshold of meaning and stop them from smoothly and automatically passing into the signified – bring to the fore the multifarious ways language is entangled with the flesh of many different kinds, which is a necessary condition for being a body word.

However, examples from the fourth section do not possess additional, sufficient reason for being body words, and that is to also attest to the way the material, as the corporeal, is vilified and cast as specifically female. Our full-throttled body words do exactly that, even when, as with the “travelling bruise”, they do not seem to at first glance. By encapsulating Cromwell’s life so perfectly in that insult, Mantel was able to show on the lexical level how the life of a commoner who rose so high in the ranks was undone by the same hierarchy – and its attendant, intersecting systems of surveillance and control – as the one constitutive of gender oppression.

To this day, the issues surrounding what one wears and how one dares to present are unfortunately prevalent in the malevolent policing of sexual matters, especially virulently against women, as well as anyone gender-non-conforming. Taking now to a metaphorical level the question of how one dresses and comports oneself, we may conclude by saying that *The Mirror and the Light* wears its feminist heart on its sleeve. Without being declarative, feminism is omnipresent in the work, as our picking at the incredibly fine fabric of Mantel’s text sought to exhibit.

### Ethics and consent

Ethical approval and consent were not required.

## Data Availability

No data associated with this article.

## Footnotes

^1^
Robertson, to whom all three novels are dedicated, became Mantel’s friend and provided her with ample scholarly help. “Robertson’s scholarship is, moreover, avowedly influenced by G. R. Elton’s political history, and especially by his book *The Tudor Revolution in Government* (1953), which she lauds as ‘magisterial’ in the way it argues that there occurred a profound transformation in the nature of the state during Henry’s reign.” ( 4 on page 149)

^2^
While Mantel’s novels may not be the clearest examples of historiographic metafiction, there is a case to be made for that classification as well: “Mantel unobtrusively weaves poignant references to Holbein’s paintings into the narrative, paying homage to the artist who single-handedly shaped the image(s) of the Henrician court that modern readers draw on as they seek to picture the early Tudor world in their minds. Almost inevitably, she alludes to Holbein’s famous double portrait The Ambassadors, a painting celebrated amongst other things for its uncanny anamorphic representation of a human skull, an image that, within the representational economy of the picture as a whole, self-reflexively highlights the two-dimensional illusion of painting. It is through their references to visual art that the novels come closest to employing the self-reflexive narrative strategies typical of historiographic metafiction.” ( 8 on page 542)

^3^
Robson 3 came up with this useful phrase, but in fact argues against such a view.

^4^
For the classic treatment of this difference see 11 on page 534–537.

^5^
From here on, and solely for *The Mirror and the Light* ^1^, the references will be to page numbers only.

^6^
For a contextual reconstruction of Senghor’s statement and critical appraisal of Frantz Fanon response to it, which was roughly the same as Ngũgĩ wa Thiong’o’s, ^19^ see 20 on page 54–59.

^7^
“I, for one, am absolutely against marginalization . . . that would amount to going back to this sort of absurd dream: ‘a man’s world/a woman’s world’. This is not so! This is a world of men and women where it is up to women to impose something which is their difference in the equality. This is a battle, and it will be hard but we cannot fight with our backs turned.” ( 24 on page 61–2)

^8^
As can be immediately surmised here, Cixous has intricate dealings with the Bible, for more see 29.

^9^
For more on this, see 32.

^10^
This connection is almost directly acknowledged in the following passages: “Beneath his clothes are the prints of his father’s fingers: bruises reversing nature, turning from the autumn black-purple of the elderberry to the pale yellow-white of the flowers. All his flesh is dappled with these shadows.” (360)
