# Supplementary material for: Bodily Abounds. Hilary Mantel’s The Mirror and the Light as Cixousian "Feminine Text
Source: Open Res Eur. 2026 Apr 10;5:236. Originally published 2025 Aug 8. [Version 2] doi: 10.12688/openreseurope.20790.2 (PMC12444272; doi:10.12688/openreseurope.20790.2)
Supplement: Peer review history

# Reviewer report: Reviewer response for version 2

**DOI:** 10.21956/openreseurope.24895.r72160

**Published:** 2026-04-14

**Authors:** Ambreen Salahuddin

**Affiliations:** 1 University of the Punjab Department of Gender Studies, Lahore, Punjab, Pakistan

The revised manuscript demonstrates significant improvement in clarity, and overall presentation. The language has been edited, so readability of the paper has improved. Sections are more coherent and present good arguments. And the conclusion is stronger and better aligned with the study’s focus. While, as per my earlier expectations, a distinct methodology section and a separate review of literature have not been included, this may reflect a different format or style of presenting research.

 In its current form, the paper is suitable for indexing.

If any, are all the source data and materials underlying the results available?

Partly

Is the work original in terms of material and argument?

Yes

Is the work clearly and cogently presented?

Partly

Is the argument persuasive and supported by evidence?

Yes

Does the research article contribute to the cultural, historical, social understanding of the field?

Partly

Does it sufficiently engage with relevant methodologies and secondary literature on the topic?

No

Reviewer Expertise:

gender studies, gender and literature, women's fiction and poetry. In PhD, my area of research was gender and fiction.

I confirm that I have read this submission and believe that I have an appropriate level of expertise to confirm that it is of an acceptable scientific standard.

# Reviewer report: Reviewer response for version 2

**DOI:** 10.21956/openreseurope.24895.r72163

**Published:** 2026-04-13

**Authors:** David Sigler

**Affiliations:** 1 University of Calgary, Calgary, Canada

My congratulations to the author Ante Andabak on this fascinating article on Mantel's *The Mirror and the Light*. The revised version works very well indeed, and is, I now feel, a very substantial addition to the conversation around Mantel as a feminist author and the broader conversation about "body words" and écriture féminine. It's exciting to see Cixous's most celebrated essay pressed into service in such an up-to-date and bold way, as a way of thinking about very recent fiction. What I especially admire is the way that Andabak captures, through Cixous's thought, the real ambivalence at the heart of Mantel's text -- this forestalls gnawing concerns about Cixous's essay being over-celebratory or possibly essentialist.

 The essay is considerably expanded since the first version, and the new elements are working very well. The response to Barthes is so fulsome, and pitched in a generatively feminist direction -- this is a really nice addition to this in terms of capturing what's at stake in the shift into "women's writing" as a matter of style rather than authorial gender identity.

 The addition of new examples of "body words" at a couple of key points also responds beautifully to my previous concerns about a certain selectivity of evidence in the prior version. In terms of textual evidence of the claims here, the expanded version works very well, and has the balance just right. I approve of the author's decision not to overhaul the order of the argument, as I had previously suggested, to start with the analysis of "gash." I think the current order, with the supplemental material coming in sections 2 and 5, as well as the conclusion, now works very well as a structure.

 A fascinating analysis of Mantel's writing as such, and an important contribution to the study of historical fiction from a feminist perspective. I am glad to have read it in its updated form! Very good work here by the author.

If any, are all the source data and materials underlying the results available?

No source data required

Is the work original in terms of material and argument?

Yes

Is the work clearly and cogently presented?

Yes

Is the argument persuasive and supported by evidence?

Partly

Does the research article contribute to the cultural, historical, social understanding of the field?

Yes

Does it sufficiently engage with relevant methodologies and secondary literature on the topic?

Yes

Reviewer Expertise:

British Romanticism, feminist theory, poststructuralism

I confirm that I have read this submission and believe that I have an appropriate level of expertise to confirm that it is of an acceptable scientific standard.

# Reviewer report: Reviewer response for version 1

**DOI:** 10.21956/openreseurope.22494.r58812

**Published:** 2025-09-17

**Authors:** Roxana Marinescu

**Affiliations:** 1 Bucharest University of Economic Studies, Bucharest, Romania

The article “Bodily abounds. Hilary Mantel’s *The Mirror and the Light *as Cixousian 'feminine text’” focuses on an original analysis of the third volume of Thomas  Cromwell trilogy by Hilary Mantel. The analysis is done from a gender perspective, based on *The Laugh of the Medusa* by Hélène Cixous (1975, Engl. transl. 1976). In her text, Cixous urges women to write themselves in, especially by using their bodies, as a means of liberation from patriarchy and, in the process, to reclaim their desires and identities. In writing themselves in, women would gain power and inspiration. In their article, Ante Andabak makes the argument that Mantel’s style corresponds to Cixous’ *écriture feminine* (and not so much the writer’s self-proclaimed gender or the focus on the main female characters). In this way, Cixous’ direction is followed, who in *The Laugh of the Medusa* was giving Jean Genêt, besides Colette and Marguerite Duras, as an example of *écriture feminine*/feminine writing.

 After an incursion into the history at the basis of the trilogy by focusing on the deeds an biography of Thomas Cromwell, the main character, at the court of Henry VIII, which constitutes the first part of the article (entitled “Properly political historical fiction”), and in which the author provides sufficient evidence of Mantel’s context in writing historical fiction, Andabak proceeds to discuss Cixous’ theoretical framework (“Cixous and the gender of writing”) as it is found in *The Mirror and the Light*. Thus, examples of Mantel’s feminine writing on the stylistic level are brought forward, even (and especially) in the analysis of Cromwell’s words, in whose mouth we find what Cixous prompted women to do, i.e. to return to their bodies, so far suppressed to them. The examples provided (scenes that capture the essence of being a woman in that epoch, i.e. giving birth) are relevant for the analysis and sufficient to allow for such an interpretation (of a feminine style in the description of Cromwell). “Body words” is the third part of the article, in which Andabak starts from Cixous’ description of women (patients in Freud’s work) who speak with their body, as language is denied to them in a process of marginalization that Cixous refers to with the metaphoric “dark continent”. In the final part of her analysis (“Travelling Bruise”), Andabak examines how corporeality is part of women writing themselves in, by examining how the word “gash” is used in Mantel’s novel and its relevance to feminine writing.

 This original analysis of Mantel’s novel is done with the support of an impressive number of secondary sources, masterly used by Ante Adabak – Cixous’ own writings[1], a number of theoretical writings on *The Laugh of the Medusa **[2]***, as well as other articles and volumes on Mantel’s work[3] and historical fiction[4], historiography (or historiographic metafiction)[5], literary theory[6], religion studies[7] and feminism[8]. To these, Mantel’s own writings are examined[9]. The interdisciplinary approach includes a literary and cultural (historical, religious) analysis, as well as elements from psychoanalysis and gender studies and feminism, which are all aptly mastered by the author in an elegant English language (although at times a little too complex to be followed with ease by a general audience). All in all, we can say that this article brings a relevant contribution to the understanding of both Hélène Cixous’ theory and Hilary Mantel’s approach to historical fiction, as well as the link between the two (which proves the initial hypothesis of the article).

 [1] The Laugh of the Medusa, as well as the article Castration or Decapitation, and the volume White ink: interviews, text and politics, The selected plays of Hélène Cixous.

 [2] Hanrahan, M: Time to Laugh or to Cry? ‘Le Rire de La Méduse’ after 40 Years, Varino, S: Liminal politics: performing Feminine difference with Hélène Cixous, Goh, I: The passion according to Cixous: from human blindness to ‘Animots’, Hanrahan M: Cixous’s Portrait de Dora, or cooking the books of Psychoanalysis

 [3] Johnston, AJ: Hilary Mantel, the Thomas Cromwell Trilogy (2009-)

 [4] Hart, M: Extraterritorial: A Political Geography of Contemporary Fiction, Duffy, E: A people’s tragedy - studies in reformation, Weigel, G: ‘Wolf Hall’ and Upmarket Anti-Catholicism. First Things, Lesser, W: Private Tudors 2024, Wood J: Invitation to a Beheading,

 [5] Hutcheon, L: Historiographic metafiction parody and the intertextuality of history, Khanna, R: Dark continents: psychoanalysis and colonialism, Gordon, LR: What Fanon said, O’Connor, S: History, Nation and Self: Wolf hall and the machinery of memory.

 [6] Auerbach, E: Mimesis: the representation of reality in western literature, Thiong’o, N: Globalectics: Theory and the Politics of Knowing, Lakoff, G, Johnson, M: Metaphors We Live By, Lakoff, G, Johnson M: Philosophy in the flesh: the embodied mind and its challenge to western thought, Gibbs, RW: Embodiment, , JT: Philology of the flesh,

 [7] Rubin, M: Corpus Christi: the Eucharist in Late Medieval Culture, Ehrman, BD: How Jesus became god: the exaltation of a Jewish preacher from Galilee

 [8] Brown, WL: Manhood and politics: a feminist reading in political theory

 [9] A memoir of my former self, Wolf Hall, What did her neighbours say when Gabriel had gone?

If any, are all the source data and materials underlying the results available?

Yes

Is the work original in terms of material and argument?

Yes

Is the work clearly and cogently presented?

Yes

Is the argument persuasive and supported by evidence?

Yes

Does the research article contribute to the cultural, historical, social understanding of the field?

Yes

Does it sufficiently engage with relevant methodologies and secondary literature on the topic?

Yes

Reviewer Expertise:

Literary studies, gender studies, feminism

I confirm that I have read this submission and believe that I have an appropriate level of expertise to confirm that it is of an acceptable scientific standard.

# Reviewer report: Reviewer response for version 1

**DOI:** 10.21956/openreseurope.22494.r58811

**Published:** 2025-09-12

**Authors:** Anwar Efendi

**Affiliations:** 1 Universitas Negeri Yogyakarta, Yogyakarta, Indonesia

In the first part (background), it is necessary to enrich the description of a number of previous studies that are relevant and up-to-date, in order to clarify the originality and novelty of the aspects studied. What is the important thing that is used as a justification (rationality) of this focus is worthy and important to study.

 The focus of the problem to be studied --- needs to be clearly formulated (it can be described in the background subsection) so that there is consistency between several aspects: supporting theory, methods used, presentation of research results, discussion of research results, and conclusions (in the article the conclusion subsection is not yet clear). 

 Conclusions (not yet clearly displayed) -- enriched with the implications of the findings so that the contribution of research results to cultural understanding and scientific development is more measurable and comprehensive

 In the first part (background), it is necessary to enrich the description of a number of previous studies that are relevant and up-to-date, in order to clarify the originality and novelty of the aspects studied. What is the important thing that is used as a justification (rationality) of this focus is worthy and important to study.

If any, are all the source data and materials underlying the results available?

Partly

Is the work original in terms of material and argument?

Partly

Is the work clearly and cogently presented?

Partly

Is the argument persuasive and supported by evidence?

No

Does the research article contribute to the cultural, historical, social understanding of the field?

Partly

Does it sufficiently engage with relevant methodologies and secondary literature on the topic?

Partly

Reviewer Expertise:

Language and Literature

I confirm that I have read this submission and believe that I have an appropriate level of expertise to confirm that it is of an acceptable scientific standard, however I have significant reservations, as outlined above.

# Reviewer report: Reviewer response for version 1

**DOI:** 10.21956/openreseurope.22494.r58133

**Published:** 2025-09-08

**Authors:** Ambreen Salahuddin

**Affiliations:** 1 University of the Punjab Department of Gender Studies, Lahore, Punjab, Pakistan

Methodology of the paper is not properly explained and there is no separate section of review of literature to know the gap in previous research and understand where this study may be placed. 

 Sentences are very long and compromise readability. Article is studded with many mistakes of grammar and language. Careful language editing is required if this must publish.

If any, are all the source data and materials underlying the results available?

Partly

Is the work original in terms of material and argument?

Yes

Is the work clearly and cogently presented?

Partly

Is the argument persuasive and supported by evidence?

Yes

Does the research article contribute to the cultural, historical, social understanding of the field?

Partly

Does it sufficiently engage with relevant methodologies and secondary literature on the topic?

No

Reviewer Expertise:

gender studies, gender and literature, women's fiction and poetry. In PhD, my area of research was gender and fiction.

I confirm that I have read this submission and believe that I have an appropriate level of expertise to state that I do not consider it to be of an acceptable scientific standard, for reasons outlined above.

# Reviewer report: Reviewer response for version 1

**DOI:** 10.21956/openreseurope.22494.r58808

**Published:** 2025-09-05

**Authors:** David Sigler

**Affiliations:** 1 University of Calgary, Calgary, Canada

This is a meaningful and interesting feminist analysis of *The Mirror and the Light* (2020), a historical novel by Hilary Mantel. The article acknowledges the novel's feminist content, but then compellingly makes the argument that it pursues a different sort of feminism meanwhile-- a feminism to be found at the level of style: "“it is, for all that, in the indomitable style of the novels … that the richest vein of feminism resides.”

 In my view, this analysis succeeds in three main ways. First, it encourages us to think about feminism as a mode of representation, a type of writing, rather than a directly political aspiration or outcome, or a subject matter. That is useful and very much in the Cixousian tradition. Second, it offers a powerful defense of Cixous's ongoing relevance for contemporary literary criticism, re-examining her best-known and oft-anthologized essay, "The Laugh of the Medusa," and defending it against claims that it is essentialist, narrow, Eurocentric, or outdated. While I don't think this essay will put those concerns to bed once and for all--e.g., in its invocations of Africa and Blackness as discussed in this essay, "The Laugh of the Medusa" might be seen to be indulging in cultural appropriation just as easily as pioneering a mode of intersectional study or Afrocentrism-- nevertheless, this does represent a highly worthwhile defense of Cixous's classic work, in a way that might stimulate a further, welcome return to this type of literary analysis. Third, the essay's strength is in its close reading of Mantel's literary language, and in staying close to the text and tracing its ambiguities and tropes, the author shows Mantel to be a brilliant writer on several levels, and that is valuable too.

 While some of the close reading is focused on imagery of mirrors and fires in the text, the core of the argument focuses on Mantel's use of "body words," or "the words exposing that we are all flesh, while also speaking to the fact that only women get fixed in it and solely perceived like that." That is, the author is endeavouring to show that Mantel's writing attends to the body and the burden that it represents for women culturally, through its materiality. Perhaps there is something ironic about this happening so thoroughly through signifiers rather than matter, but I do find it to be a quite insightful argument. The argument focuses especially on a single short sentence from *The Mirror and the Light*, in which she uses the word "gash." Andabak shows the different uses that this word is asked to perform in this novel, from wounds to genitals to a metonymous antifeminist slur. Even as Mantel mostly uses the term in its most literal sense, Andabak suggests that the word continues to carry these other meanings and, through that process, links the court of Henry VIII to bodies and to antifeminist epistemic violence.

 The other important example of analysis of body words here is the focus on Mantel's diction as surrounding Catholicism, and especially the Virgin Mary. Andabak shows that the question of Mary's virginity arises especially through a thicket of translation issues, which puts the cultural issues at the heart of the novel at the level of signification and specifically "body words" as a cultural inheritance. This I find to be a very effective argument.

 These are effectively argued, I believe. Yet they are very slight examples -- we are talking about just a few sentences out of a monumental huge novel, which is the third of a momumental huge trilogy. At times I wish for more evidence, then, to support the claims being made here. I would encourage the other to find at least one more example of a clear "body word" from *The Mirror and the Light* and to analyze it for a paragraph or so, just to show that this is a *pattern* throughout the novel. Because any novel this large is bound, even if just inadvertently, to use body-related words in a way that might have some polysemy to it-- that's just the nature of language. A second or second-and-third example of Mantel doing this would make the argument more compelling, I feel.

 Beyond that, my main advice for this essay would be to restructure its argument, to make the stakes more apparent and to root the question in an interpretation of Mantel's text rather than an application of Cixous's. I would suggest that the argument would be more persuasive if it could be flipped around, effectively, or inverted. That is: I would suggest that the essay start with an analysis of the word "gash" in Mantel's text and the discussion surrounding virginity and translation, as these are the core evidence for the argument. Then I would open into: well, what is going on here? what does this mean? and to answer that, I would propose that Mantel may be using the strategies of écriture feminine, in the way that Cixous famously discusses. From there, I would indicate (as you are now doing near the beginning of this current version) that this presence of these body words (as such) ought to complicate our understanding of the novel's feminism-- because the feminism, which is already readily apparent in the novel's content, is also there, and differently there, in its style. From there, I would make the case (as you do near the start of this version) that, through her use of écriture feminine, Mantel is remaking the genre of historical fiction, from something middlebrow and reassuring into something dangerous and modernist. This is the same argument you are already making in this version-- it's just basically reversing the order, so that the interpretation of the novel is the foundation of it, rather than an afterthought, and the consequences of your analysis can arise from that analysis, rather than being a preface to the analysis.

 If revised in this way, you would be "discovering" écriture feminine in Mantel and then seeking to explain its significance, rather than presenting it as a methodological decision of yours. You could then focus the second half of the essay on the significance of this type of feminism for our understanding of Mantel's writing, the Cromwell trilogy, or historical fiction in general.

 Additionally, there are things about Cixous's essay that I would like to see explained more fully and deeply here. First, I would like to have a deeper sense from Cixous of what the criteria are for "body words," because she writes so metaphorically. If we cannot rely on the gender identification of the author for guidance (e.g., in the case of Jean Genet), how do we know when we have encountered words that pertain to the body in a "feminine" way? My concern is that, because nearly every long text will inevitably use words related, at least tangentially, to the body, it may be possible to assert that absolutely any text is écriture feminine, if we like it! I just want a clearer sense of what the criteria are in your view for écriture feminine, to make it clearer it's not just a term we use for "writing we like and want to praise." If we are looking for writing that is, as Cixous says, "very close to the voice," wouldn't any use of direct dialogue, or onomatopoeia, constitute écriture feminine? 

 Second: the author might also, briefly, situate Cixous and Mantel in relation to another classic text often featured in introductory theory courses -- "The Death of the Author" by Roland Barthes. I am thinking of the very opening of that essay, where he asks "Who is speaking in this way" in his brief analysis of a passage from Balzac. His argument that "it will always be impossible to know" might help the author reframe a similar dilemma confronted in this article: “While the thoughts recorded are of Cromwell, the character – there are no narratorial asides or metalepses in the novels – their virtuoso renderings in sentences are decidedly authorial, without being anachronistic.”

 Third: at one point in the essay, the author seeks to demonstrate that Cixous was an early champion of what is today called "embodied cognition," and seeks to validate Cixous's argument based on the subsequent findings of cognitive scientists. If the author wishes to make this move--which is a worthwhile one, I think--they would need to show that cognitive scientists have been similarly willing to place embodied cognition under the sign of the feminine. That is, to really make the connection to Cixous, you would want to show how cognitive scientists are taking into account sexual difference or gender when arriving at their conclusions about embodied cognition.

 Fourth: and this is just a matter of style, and could be easily cleaned up: the author argues that Mantel will use "run of the mill idioms" to literalize them. Given that this is an important part of the argument, the author will want to be very purposeful in their own use of similar idioms. There is a tendency sometimes to clichéd phrases in this essay that I am not sure are intentionally there to do this type of literalizing work: e.g. "“take a stab at fleshing out”, “chockful of scenes.”

 Finally, just to note a few small typographical errors, so they can be fixed in the next version: 

 “Crucible in which the modern English state” – is there a missing the at beginning? Also, maybe better to avoid the cliché “crucible in which X was formed” formulation, per note just above. The sentence as it currently stands is also odd because it goes on the say “wielded” – this to me seems to be a mixed metaphor, in the sense that crucibles are, when used, too hot to pick up with the hands—they are usually picked up with tongs, and thus never exactly a thing “wielded.”

 Also there are small typos in: “more determined by **bodily** than men”; “unreasonable and dead wrong to **upheld** the opposition.”

 Overall, I found this to be a highly interesting essay and a worthwhile analysis of the Mantel novel. It is great to see new work in the classic Cixousian vein, using "Laugh of the Medusa" as a way into thinking about very recent feminist literature at the level of its style rather than content. That is well done and a highly engaging argument, I feel.

If any, are all the source data and materials underlying the results available?

No source data required

Is the work original in terms of material and argument?

Yes

Is the work clearly and cogently presented?

Yes

Is the argument persuasive and supported by evidence?

Partly

Does the research article contribute to the cultural, historical, social understanding of the field?

Yes

Does it sufficiently engage with relevant methodologies and secondary literature on the topic?

Yes

Reviewer Expertise:

British Romanticism, feminist theory, poststructuralism

I confirm that I have read this submission and believe that I have an appropriate level of expertise to confirm that it is of an acceptable scientific standard, however I have significant reservations, as outlined above.
